# Therapeutic Effect of Biejiaxiaozheng Pills on Carbon Tetrachloride-Induced Hepatic Fibrosis in Rats through the NF-*κ*B/Nrf2 Pathway

**DOI:** 10.1155/2021/3954244

**Published:** 2021-11-24

**Authors:** Weibin Wu, Liqiang Li, Jian Yang, Pinyu Li, Yuying Hu, Guifeng Zhang, Xiaozhong Zhu

**Affiliations:** ^1^Zhaoqing Medical College, Zhaoqing 526020, China; ^2^Zhaoqing First People's Hospital, Zhaoqing 526020, China; ^3^Zhaoqing Hospital of Traditional Chinese Medicine, Zhaoqing 526020, China

## Abstract

**Aims:**

To explore the effects of Biejiaxiaozheng pills on carbon tetrachloride-induced hepatic fibrosis in rats through the NF-*κ*B/Nrf2 pathway and to explore the possible antifibrotic mechanisms of the drug. *Material and Method.* A rat model of hepatic fibrosis was established via CCl_4_ induction. Liver function and antioxidant indices were detected using commercial kits. Hematoxylin-eosin and Masson staining were used to detect pathological changes in hepatic tissues. ELISA was used to measure plasma TNF-*α*, IL-*β*, and IL-6 levels. RT-PCR was used to measure changes in TNF-*α*, IL-*β*, and IL-6 levels in hepatic tissues. Changes in p65, P-p65, Nrf2, and HO-1 protein expression were detected using western blotting.

**Results:**

In rats with hepatic fibrosis, Biejiaxiaozheng pills effectively improved liver function, alleviated fibrosis in hepatic tissues, and significantly reduced collagen accumulation. The pills significantly downregulated inflammatory cytokine expression in hepatic tissues by suppressing p65 phosphorylation and reduced plasma inflammatory cytokine levels to some extent. The pills upregulated Nrf2 and HO-1 expression in hepatic tissues, enhanced antioxidant potential, and upregulated plasma antioxidant levels.

**Conclusion:**

Biejiaxiaozheng pills improved hepatic fibrosis symptoms and lesions in rats, likely by inhibiting the NF-*κ*B pathway and promoting the Nrf2 pathway.

## 1. Introduction

Hepatic fibrosis is a type of hepatic pathological change induced by a long-term chronic injury of hepatocytes due to diseases such as nonalcoholic fatty liver disease and hepatitis B, and its main manifestation is the excessive deposition of extracellular matrix in hepatocytes [1, 2]. Hepatic fibrosis mainly occurs due to the activation and proliferation of hepatic stellate cells under the regulation of cytokines secreted by Kupffer cells, which enhances the synthesis of extracellular matrix and reduces its degradation, thus gradually leading to fibrosis formation [3, 4]. Fibrosis can be effectively reversed if drug intervention is administered on time during the occurrence/progression of hepatic fibrosis [5, 6]. During this process, Kupffer cells are activated by external stimuli to secrete cytokines, and they further activate hepatic stellate cells via paracrine effect. Following this, the activated hepatic stellate cells can recruit a large number of Kupffer cells through similar paracrine effects. This cycle is maintained for a long period and continuously promotes hepatic fibrosis [7–9]. Many studies have proved that inflammatory factors and reactive oxygen species play important roles in the aforementioned positive feedback cycle. Therefore, if drugs can improve the functions of the NF-*κ*B and Nrf2 pathways, they can produce some therapeutic effects against hepatic fibrosis [10, 11].

Biejiaxiaozheng pills are a compound preparation comprising Carapax Trionycis, *Scirpus fluviatilis*, *Curcuma zedoaria*, *Panax quinquefolius*, *Astragalus propinquus Schischkin*, *Paeonia sterniana*, *Atractylodes macrocephala* Koidz, *Wolfiporia extensa* Ginns, *Rheum rhabarbarum*, *Glycyrrhiza uralensis*, and synthetic musk. Basic research has shown that the aforementioned components show some antifibrotic effects [12, 13]. As a fixed compound preparation, Biejiaxiaozheng pills have been used in clinical practice for many years, and these pills have been confirmed to be effective in the treatment of hepatic fibrosis through clinical and experimental studies [14, 15]. In the present study, a rat model of hepatic fibrosis was established via carbon tetrachloride (CCl_4_) induction to study whether Biejiaxiaozheng pills can reverse hepatic fibrosis by improving the functions of the NF-*κ*B and Nrf2 pathways.

## 2. Materials and Methods

### 2.1. Materials

Biejiaxiaozheng pills (3.6 g/pill) and propranolol (10 mg/pill) were purchased from Zhaoqing Hospital of Traditional Chinese Medicine. Sprague Dawley rats and a high-fat diet were purchased from the Laboratory Animals Center of Hubei Disease Prevention and Control Center (China). CCl_4_ and TBST with Tween 20 (powder) were purchased from Shanghai Macklin Biochemical Co., Ltd. (Cat. No. C805332, T854550, China). PVDF membrane was purchased from Millipore Inc. (Cat. No. IPVH00010, USA). Aspartate aminotransferase (AST) and alanine aminotransferase (ALT) detection kits, total protein detection kit, albumin detection kit, and total bilirubin detection kit were purchased from Nanjing Jiancheng Bioengineering Institute (Cat. No. C010-2-1, C009-2-1, A045-2-2, A028-2-1, C019-1-1, China). TNF-*α*, IL-1*β*, and IL-6 ELISA kits were purchased from Dakewe Biotech Co., Ltd. (Cat. No. 1317202, 1310122, 1310602, China). Total RNA rapid extraction kit was purchased from Beijing Tianmo Biotech Co., Ltd. (Cat. No. TR205, China). Total SOD activity detection kit, lipid peroxidation (MDA) detection kit, total glutathione (GSH) detection kit, glutathione peroxidase (GPx) detection kit, SYBR Green qPCR Mix (High ROX), cDNA synthesis reagent kit, DEPC water, BCA protein concentration detection kit, western and IP cellular lysis buffer, protease inhibitor cocktail, SDS-PAGE protein loading buffer, SDS-PAGE gel rapid preparation kit, electrophoresis buffer, transfer buffer, PVDF membrane activation buffer, prestained protein molecular weight standards, dry BSA powder, and ultrasensitive ECL chemiluminescence detection kit were purchased from Beyotime Institute of Biotechnology (Cat. No. S0101, S0131, S0052, S0056, D7265, D7170, R0022, P0010, P0013, P1006, P0286, P0012AC, P0562, P0575, P0021S, P0077, ST023, P0018AS, China). The primary antibodies against p65, P-p65, Nrf2, HO-1, and *β*-actin as well as HRP-labeled goat anti-rabbit IgG secondary antibodies were purchased from Affinity Biosciences Co. Ltd. (Cat. No. AF5006, AF2006, AF0639, AF5393, AF7018, S0001, USA).

### 2.2. Establishment of Hepatic Fibrosis Rat Model and Treatment

A CCl_4_/olive oil solution (50% (*v*/*v*)) was administered intragastrically twice a week (Monday and Thursday) at 0.2 mL/100 g body weight, combined with high-fat and high-ethanol diets for induction. Distilled water and normal feed were administered to the control group, and the corresponding weight of olive oil solution was administered intragastrically. In the 12^th^ week, three control rats and three model rats were randomly selected. Following ethyl carbamate anesthesia, blood samples were collected from the inferior vena cava to measure ALT and AST levels, and hepatic tissues were stained with HE and Masson staining. Indexes of the control and model groups were compared to determine the successful establishment of the model. After the model was successfully constructed, the surviving rats were divided into the CCl_4_ group, CCl_4_+propranolol (10 mg/kg) (CCl_4_+PRO) group, CCl_4_+Biejiaxiaozheng pill (0.6 g/kg) (CCl_4_+BJXZ0.6) group, CCl_4_+Biejiaxiaozheng pill (1.2 g/kg) (CCl_4_+BJXZ1.2) group, and the control group (8 rats/group) for treatment. Method of drug administration: modest doses of drugs were weighted, and the suspension is prepared in a mortar. The concentration of propranolol was 5 mg/mL, and Biejiaxiaozheng pills were 0.24 g/mL, respectively. Each group was intragastric administration one time every day.

### 2.3. Detection of Rat Body Weight

A 1 L beaker was placed on the electronic balance. After taring, the beaker was removed and the rat was placed in the beaker before the beaker was placed on the balance. The reading was obtained after the rats had calmed down.

### 2.4. Detection of Hepatic Function Markers

Following ethyl carbamate anesthesia, the abdomen was opened and blood was collected from the inferior vena cava using heparin as an anticoagulant. Then, the blood sample was analyzed according to the instructions of the kits, and the test results were converted into enzyme activity units according to the instructions.

### 2.5. HE and Masson Staining of Hepatic Tissue

The rat liver was fixed in 4% paraformaldehyde for >24 hours and then embedded in paraffin to prepare paraffin sections. HE and Masson staining were used to detect the degree of tissue injury and fibrosis.

### 2.6. Measurement of TNF-*α*, IL-1*β*, and IL-6 Levels

After ethyl carbamate anesthesia, blood was collected from the inferior vena cava of rats and anticoagulated with heparin. According to the requirements of the ELISA kit, the blood samples were centrifuged at 3000 rpm for 15 min at low temperature, and the supernatant was obtained to analyze the blood samples according to the instructions.

### 2.7. Measurement of SOD, MDA, GSH, and GPx

After ethyl carbamate anesthesia, blood was collected from the inferior vena cava. After sample collection and processing according to the manufacturer's instructions, the blood samples were analyzed.

### 2.8. RT-PCR

The total RNA extraction kit was used to extract RNA from rat liver. The extracted RNA was reverse transcribed into cDNA using a cDNA synthesis kit. Transcripts of the inflammatory factors TNF-*α*, IL-1*β*, IL-6, and *β*-actin were detected by the SYBY GREEN detection kit. *β*-Actin transcript level was used as a reference for calculating the relative expression levels of various genes, expressed as the RQ value. The primer sequences used are shown in Table 1.

### 2.9. Western Blotting

Appropriate amount of liver tissue was weighed, and the total protein was extracted and quantified using a kit. The protein samples were homogenized and subjected to gel electrophoresis. Then, the proteins were transferred to a PVDF membrane via the wet method and blocked with 5% BSA for 1 hour before incubation with primary antibodies at 4°C overnight. After incubation, the membrane was washed with TBST four times and incubated with secondary antibodies at 37°C for 45 minutes. The membrane was washed with TBST four times to remove the secondary antibodies. Finally, the protein bands were developed, and the images were collected using an ECL photoluminescence solution and a gel imaging system. Relative expression of each protein was expressed as the ratio of the target protein/internal reference protein.

### 2.10. Statistical Analysis

ImageJ was used for semiquantitative analysis of HE staining, Masson's trichrome staining, and protein bands. SPSS 25 was used for statistical analysis. GraphPad was used to generate graphs. One-way ANOVA was used to analyze differences among groups. A difference of *P* < 0.05 was considered statistically significant.

## 3. Results

### 3.1. Effects of BJXZ Pills on Body Weight of Rats with Hepatic Fibrosis

**Figure 1:**
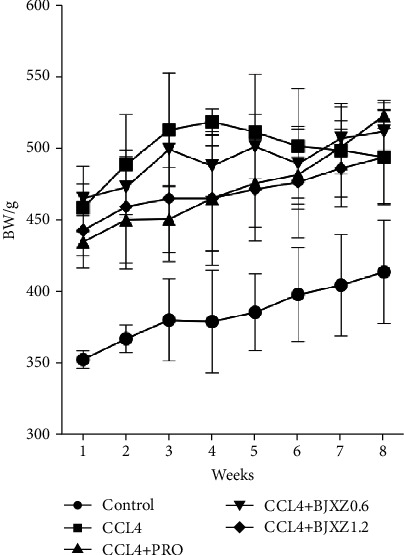
Effects of BJXZ pills on rat body weight. Data are shown as the mean ± SD.

After the model construction was complete, the weights of the model rats were significantly higher than those of control rats. With the progress of treatment, the body weights of rats in the model group continued to rise and began to decrease after the fourth week of treatment. The body weight of rats in each treatment group and the control group continued to increase during the treatment period of 8 weeks. Therefore, the drug may have produced a certain reversal effect on weight loss caused by the progression of hepatic fibrosis in rats. Among the treatment groups, the weight increase in the propranolol group was the most significant (Figure 1).

### 3.2. Effects of BJXZ Pills on Liver Function in Rats with Hepatic Fibrosis

**Figure 2:**
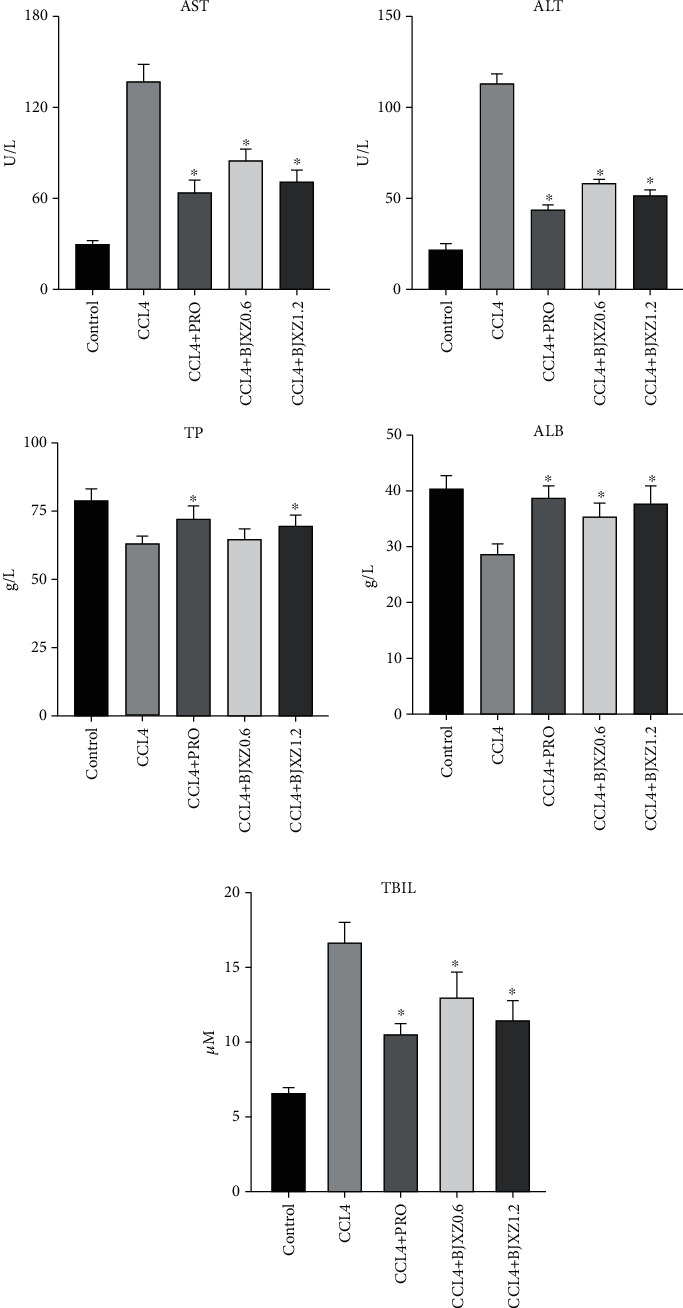
Improvement effects of BJXZ pills on liver function in rats. Data are shown as the mean ± SD (^∗^vs. control, ^∗^*P* < 0.05, *n* = 8).

Compared with those in the control group, blood ALT, AST, and total bilirubin levels were significantly increased, but total protein and albumin levels were significantly decreased in the CCl_4_ group. Biejiaxiaozheng pills significantly reversed changes in the CCl_4_ group. And the high-dose Biejiaxiaozheng pills responded equally to propranolol (Figure 2).

### 3.3. Effects of BJXZ Pills on Fibrosis in Hepatic Tissues

**Figure 3:**
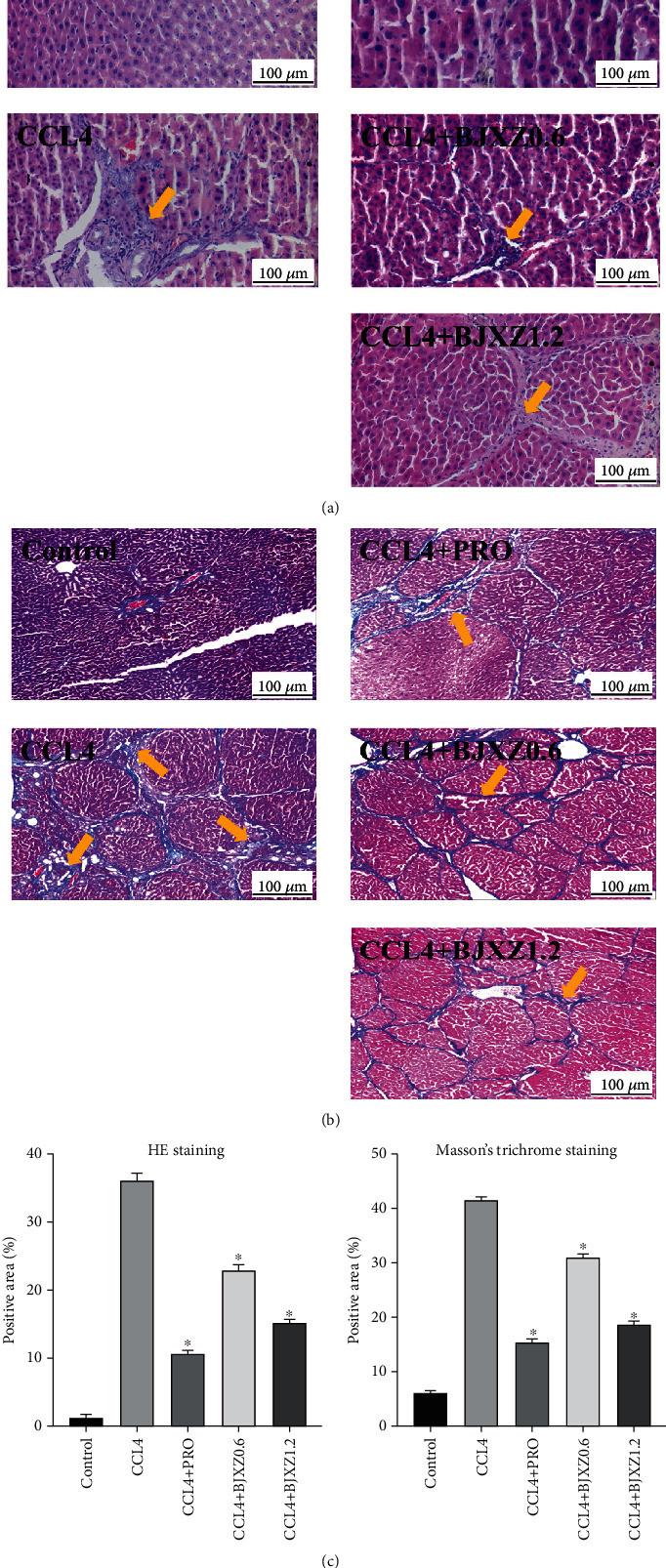
Improvement effects of BJXZ pills in rats with hepatic fibrosis: (a) hematoxylin and eosin staining (×40); (b) Masson's trichrome staining (×40); (c) morphometric analysis of HE staining and Masson's trichrome staining. Data are shown as the mean ± SD (^∗^vs. control, ^∗^*P* < 0.05, *n* = 3).

In the control group, the hepatic lobule structure was normal. The hepatic cords were arranged in a regular manner, and necrosis, degeneration, and inflammatory cell infiltration were not observed. The fiber level was low. In the model group, the hepatic lobule structure was disrupted (Figure 3(a)), and the hepatic cords were arranged in a disorderly manner. Necrosis, degeneration, and inflammatory cell infiltration were observed, and interstitial hyperplasia was significant. Collagen fibers were wrapped around hepatic lobules to form apparent pseudolobules (Figure 3(b)). In the Biejiaxiaozheng pill group, the degree of pseudolobules, inflammatory cell infiltration, and necrosis were significantly improved compared with those in the model group. The therapeutic efficacy within the Biejiaxiaozheng pill group was slightly poorer than that in the propranolol group (Figure 3(c)).

### 3.4. Effects of BJXZ Pills on Blood TNF-*α*, IL-1*β*, and IL-6 Levels in Rats with Hepatic Fibrosis

**Figure 4:**
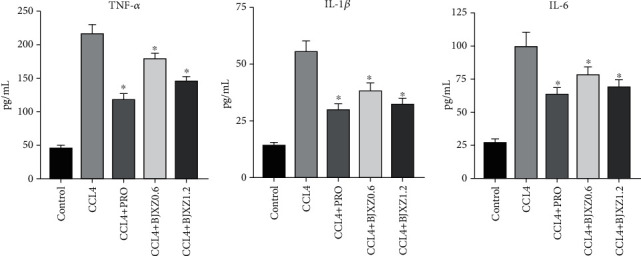
Effects of BJXZ pills on blood TNF-*α*, IL-1*β*, and IL-6 levels in rats with hepatic fibrosis. Data are shown as the mean ± SD (^∗^vs. control, ^∗^*P* < 0.05, *n* = 8).

Serum TNF-*α*, IL-1*β*, and IL-6 levels in the CCl_4_ group were significantly increased compared with those in the control group. In the Biejiaxiaozheng pill group, TNF-*α* and IL-1*β* levels were significantly decreased, and IL-6 was downregulated; however, the effect of Biejiaxiaozheng pills on TNF-*α* was significantly weaker than that of propranolol, while the effects of Biejiaxiaozheng pills on IL-1*β* and IL-6 were not significantly different from those of propranolol, with *P* values of 0.079 and 0.066, respectively (Figure 4).

### 3.5. Effects of BJXZ Pills on Hepatic Inflammatory Factor Transcript Levels in Rats with Hepatic Fibrosis

**Figure 5:**
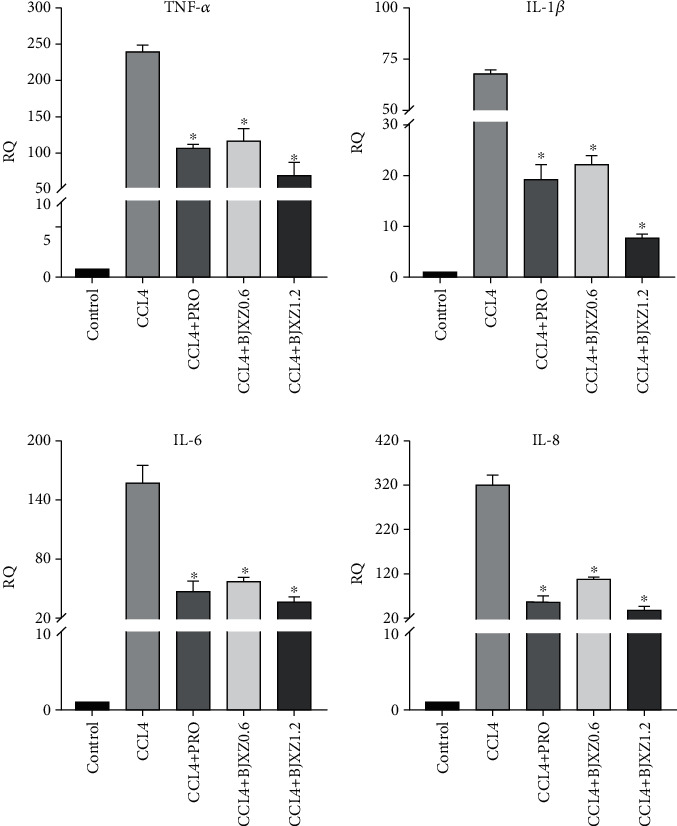
Effects of BJXZ pills on liver inflammatory factor transcript levels in rats with hepatic fibrosis. Data are shown as the mean ± SD (^∗^vs. control, ^∗^*P* < 0.05, *n* = 3).

In liver tissues, the transcript levels of TNF-*α*, IL-1*β*, and IL-6 were significantly increased in the treatment groups. Biejiaxiaozheng pills significantly reduced the transcript levels of these three inflammatory factors, and the improvement effects of Biejiaxiaozheng pills were significantly better than those of propranolol (Figure 5).

### 3.6. Effects of BJXZ Pills on Blood MDA, SOD, GSH, and GPx Levels in Rats with Hepatic Fibrosis

**Figure 6:**
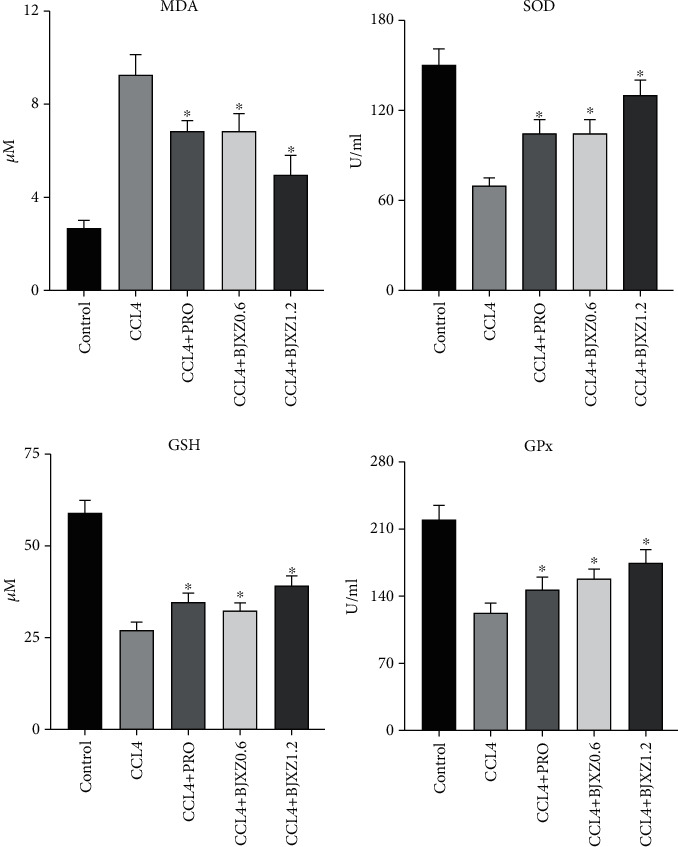
Effects of BJXZ pills on blood MDA, SOD, GSH, and GPx levels in rats with hepatic fibrosis. Data are shown as the mean ± SD (^∗^vs. control, ^∗^*P* < 0.05, *n* = 8).

In the CCl_4_ group, plasma MDA level was significantly increased, showing that the degree of lipid peroxidation was higher in rats with hepatic fibrosis. However, Biejiaxiaozheng pills significantly decreased MDA levels and improved high lipid peroxidation in rats. At the same time, SOD, GSH, and GPx levels were significantly decreased, while MDA level was significantly increased in rats with hepatic fibrosis. However, Biejiaxiaozheng pills significantly increased SOD, GSH, and GPx levels, enhancing antioxidant activity in rats (Figure 6).

### 3.7. Effects of BJXZ Pills on Nrf2, HO-1, p65, and P-p65 Expression in the Liver of Rats with Hepatic Fibrosis

**Figure 7:**
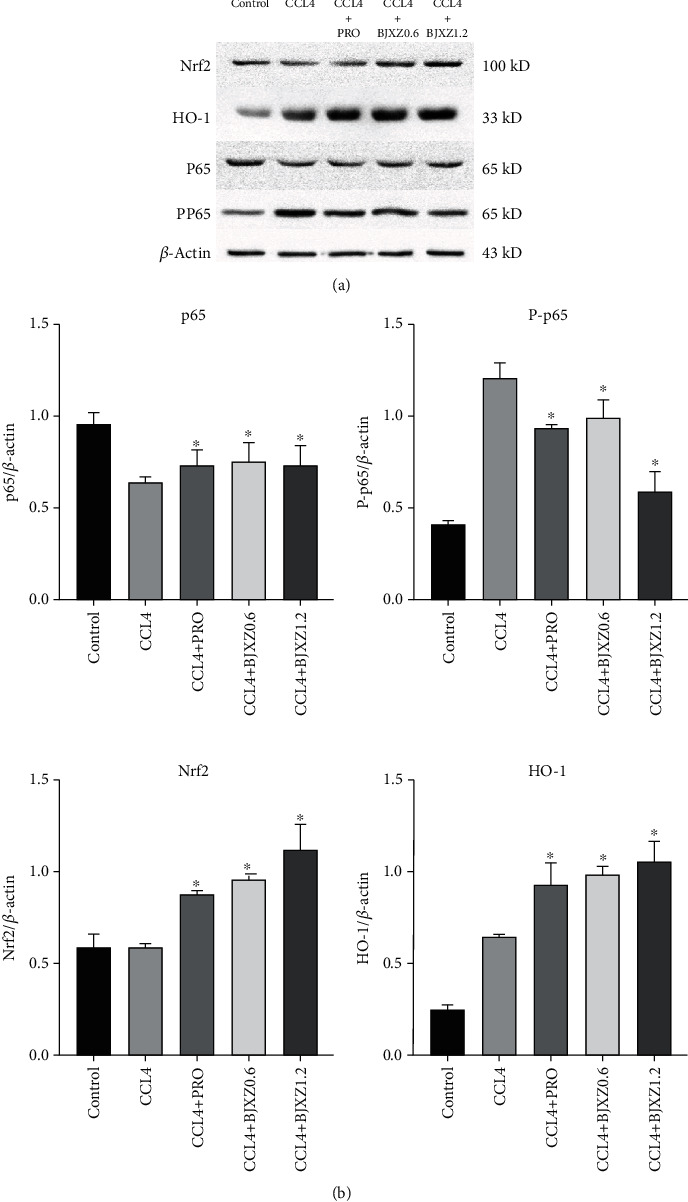
Effects of BJXZ pills on liver p65, P-p65, Nrf2, and HO-1 expression in rats with hepatic fibrosis. (a) Western blots for p65, P-p65, Nrf2, and HO-1 in liver tissue. (b) The relative optical density was normalized to *β*-actin. Data are shown as the mean ± SD (^∗^vs. control, ^∗^*P* < 0.05, *n* = 3).

Compared with the control and CCl_4_ groups, all treatment groups showed significantly upregulated Nrf2 and HO-1 expression in the Nrf2/HO-1 pathway and enhanced liver antioxidant activity; these effects were the most significant in the Biejiaxiaozheng pill group. Regarding the NF-*κ*B pathway, p65 expression in the control group was significantly higher than that in the other groups, but there were no significant differences among the CCl_4_ and other groups. P-p65 expression in the CCl_4_ group was significantly higher than that in the other groups, but P-p65 expression in each treatment group was lower than that in the control group. P-p65 expression in the Biejiaxiaozheng pill group was the lowest, which inhibited the activation of the NF-*κ*B pathway (Figure 7).

## 4. Discussion

Every year, more than 2 million people worldwide die due to cirrhosis and liver cancer caused by viral hepatitis and alcoholic fatty liver disease [16]. Hepatic fibrosis is a type of hepatic scarring reaction that occurs after hepatocytes are injured by one or more external factors. It is the intermediate stage of transformation from various chronic liver diseases to liver cirrhosis and hepatocellular carcinoma. Its occurrence and development are related to chemokines, neuroendocrine, angiogenesis, inflammation, oxidative stress, and other pathways [17, 18]. A large volume of studies showed that if drugs can regulate the NF-*κ*B and Nrf2 pathways simultaneously, hepatic fibrosis may improve [19, 20]. Moreover, there are a large number of components in herbal medicine that can regulate the above pathways simultaneously to improve pathological changes due to many types of fibrosis in the body [21, 22].

Biejiaxiaozheng pills, a traditional Chinese medicine compound preparation that has been used clinically for more than 30 years, have been found to show a certain curative effect on the hemorrhage of the digestive tract, portal hypertension, and hepatic fibrosis caused by hepatitis B and cirrhosis [14, 23]. However, the mechanism of action of this prescription has not been fully elucidated. In this study, CCl_4_, ethanol, and high-fat diet induction were used to construct a rat model of hepatic fibrosis, which was then administered Biejiaxiaozheng pills for 8 weeks to observe the therapeutic effects. At the same time, propranolol, which can decrease portal hypertension and treat hepatic fibrosis, was used as controls in this study [24, 25].

The results of this study showed that the weight of rats in the control and treatment groups gradually increased after 8 weeks of treatment. On the other hand, the weight of rats in the model group rapidly increased in the first 4 weeks before gradually decreasing in the subsequent 4 weeks. This may be due to the gradual conversion of hepatic fibrosis to cirrhosis in the model group. Liver function test results showed that liver function in the model group was significantly impaired, which presented as significant increases in plasma ALT, AST, and total bilirubin levels and significant decreases in plasma total protein and albumin levels. Conversely, transaminase and total bilirubin levels were significantly decreased in the various treatment groups, whereas plasma albumin levels were increased significantly. Plasma total protein level was significantly improved only in the propranolol and Biejiaxiaozheng pill groups.

The liver function test results preliminarily showed that Biejiaxiaozheng pills show some efficacy against hepatic fibrosis in rats. HE and Masson staining of rat livers showed that the drug significantly decreased the degree of hepatic fibrosis, which presented as a significant reduction in liver pseudolobules and a decrease in fiber content. These results show that Biejiaxiaozheng pills can alleviate hepatic fibrosis directly to achieve its therapeutic effects, although these effects are weaker than those of propranolol.

After confirming the effect of drugs on inhibiting and reversing hepatic fibrosis, we studied its possible mechanisms. Previous studies have shown that activation of the NF-*κ*B pathway and inhibition of the Nrf2 pathway play a mediating role in the occurrence and development of tissue fibrosis. The active components of propranolol and various wild plants can reverse different pathological changes by improving the function of the above pathways [26, 27]. Therefore, we explored whether Biejiaxiaozheng pills act through regulating the NF-*κ*B and Nrf2 pathways. First, the contents of inflammatory factors and antioxidant-related substances in the blood of rats were detected. The results showed that the contents of inflammatory factors such as TNF-*α*, IL-1*β*, and IL-6 and lipid peroxidation marker MDA in the model group were significantly increased but those of the antioxidants SOD, GSH, and GPx were significantly decreased. Biejiaxiaozheng pills significantly reversed these changes; however, the effect of Biejiaxiaozheng pills on inflammatory factors was weaker than that of propranolol. Moreover, the reversal effect of Biejiaxiaozheng pills was the best in terms of anti-oxidation-related indexes. In addition to blood markers, we measured the transcript levels of inflammatory factors in liver tissues. The results showed that Biejiaxiaozheng pills significantly inhibited the transcript levels of inflammatory factors in liver tissues, with the inhibitory effect on IL-6 being the most obvious.

Finally, we studied whether the drugs affected core proteins in the NF-*κ*B and Nrf2 pathways. The results showed that Biejiaxiaozheng pills significantly enhanced the expression levels of Nrf2 and HO-1 to increase the effects of the Nrf2 pathway, which further increased the antioxidant activity. At the same time, Biejiaxiaozheng pills decreased p65 phosphorylation and inhibited NF-*κ*B activation to reduce inflammation and ultimately reverse hepatic fibrosis.

In summary, Biejiaxiaozheng pills inhibit the activation of the NF-*κ*B pathway while simultaneously enhancing the effects of the Nrf2 pathway to alleviate inflammation and high oxidative stress induced by CCl_4_, thereby achieving antifibrotic effects.

## Figures and Tables

**Table 1:** Sequences of primers.

Gene	Forward	Reverse
TNF-*α*	AGAACAGCAACTCCAGAACAC	CACGAGCAGGAATGAGAAGAG
IL-1*β*	GGATGATGACGACCTGCTAGT	CACTTGTTGGCTTATGTTCTGTC
IL-6	CCAACTTCCAATGCTCTCCTAAT	CGAGTAGACCTCATAGTGACCTT
*β*-Actin	TCAGGTCATCACTATCGGCAAT	ACTGTGTTGGCATAGAGGTCTT

## Data Availability

The data used to support the findings of this study are included within the article.
